# T-3 Delayed Hospitalization and Burn Mortality: Insights from a Nationwide Retrospective Review

**DOI:** 10.1093/jbcr/irag033.003

**Published:** 2026-04-06

**Authors:** Christopher J Fedor, Mia J Carrarini, Sarah M Tepe, Mare G Kaulakis, Catherine K Perez, Jenny A Ziembicki, Francesco M Egro

**Affiliations:** University of Pittsburgh Medical Center Department of Plastic Surgery, Pittsburgh, PA; University of Pittsburgh Medical Center, Pittsburgh, PA; University of Pittsburgh Medical Center, Pittsburgh, PA; University of Pittsburgh Medical Center Mercy Burn Center, Pittsburgh, PA; University of Pittsburgh Medical Center Mercy Burn Center, Pittsburgh, PA; University of Pittsburgh Medical Center, Pittsburgh, PA; University of Pittsburgh Medical Center, Pittsburgh, PA

## Abstract

**Introduction:**

Timely access to care is a critical factor that influences outcomes in burn patients, with early intervention linked to improved survival, shorter hospital stays, and fewer complications. Delays in receiving treatment, including delays due to secondary transfers from external facilities, remain a common challenge in burn care. While the impact of delayed admission has been studied, its effects across diverse patient populations is not fully understood. This study evaluated the relationship between time to admission and outcomes, including survival rate, length of hospital stays and incidence of complications.

**Methods:**

We conducted a retrospective cohort study using American Burn Association (ABA) Burn Care Quality Platform (BCQP) registry data from 2013-2022. Patients were separated into two groups – admission within 48 hours of injury, and delayed admission within 2-14 days of injury. Data collection included demographics, time to presentation at the hospital, and outcomes, including mortality, length of hospital and/or ICU stay, infection rates, and graft/flap loss.

**Results:**

Of 266 983 burn patients, 80% were admitted within 48-hours post-injury and 20% had delayed admission. Patients with delayed admission were moderately older (41.8 ± 22.7 vs 35.0 ± 23.7, *p*>.05), had smaller TBSA (5.0 ± 8.9 vs 8.2 ± 12.5, *p*>.05), and a lower mortality rate (1.4% vs. 3.6%, *p*<.001). These patients also had a longer median hospital stay (+ 2 days, *p*<.001) but a shorter median ICU stay (0 days vs. 1 day, *p*<.001). Delayed admission was further associated with lower rates of infection, multiple organ failure, and graft/flap loss requiring further intervention (all *p*<.001).

**Conclusions:**

While delayed admission was associated with a longer overall hospital stay, these patients had shorter ICU stays and fewer complications. These findings suggest that delayed presentation may not universally predict worse outcomes. Further investigation is warranted to understand the drivers behind these differences and to optimize transfer protocols in burn care systems.

**Applicability of Research to Practice:**

The results of this study suggest that timeliness alone may not be a reliable predictor of poor outcomes in burn care. Thus, a more nuanced approach to triaging burn patients, including an emphasis on injury severity, transfer timing, and initial care quality, should be used. Future studies may focus on identifying which patient and injury factors mediate the relationship between delayed care and outcomes.

**Funding for the study:**

N/A.

